# 

**Published:** 1895-10-19

**Authors:** 


ABSOLUTELY PURE, therefore BEST.

**CADBURY'S COCOA**

Of absolute purity and freedom from alkali." —*Braithwaite.*

**CADBURY'S COCOA**

"The Typical Cocoa of English Manufacture—Absolutely Pure."—*The Analyst.*

Index S. G. O.

### THE HOSPITAL

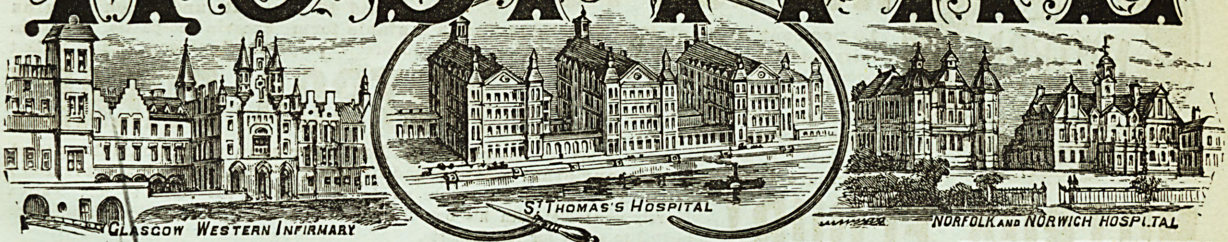

No. 473. Vol. XIX.

SATURDAY, OCT. 19TH, 1895.

**WITH EXTRA NURSING SUPPLEMENT.**

[Registered at the General Post Office as a Newspaper for transmission in the United Kingdom and Abroad.]

